# Supplementary material for: A Critical View of the Application of the APEX Software (Aqueous Photochemistry of Environmentally-Occurring Xenobiotics) to Predict Photoreaction Kinetics in Surface Freshwaters
Source: Molecules. 2019 Dec 18;25(1):9. doi: 10.3390/molecules25010009 (PMC7017383; doi:10.3390/molecules25010009)
Supplement: Supplementary file 1 [file molecules-25-00009-s001.zip › 3.2.4_gcc-4.4.0.zip!/3.2.4_gcc-4.4.0/3.2.4_gcc-4.4.0/doc/octave/html/interpreter/Multiple-Plots-on-One-Page.html]

Multiple Plots on One Page - Untitled


Next: Multiple Plot Windows,
Previous: Plot Annotations,
Up: Plotting Basics


---

#### 15.1.4 Multiple Plots on One Page

Octave can display more than one plot in a single figure. The simplest
way to do this is to use the `subplot` function to divide the plot
area into a series of subplot windows that are indexed by an integer.
For example,

```
     subplot (2, 1, 1)
     fplot (@sin, [-10, 10]);
     subplot (2, 1, 2)
     fplot (@cos, [-10, 10]);
```

creates a figure with two separate axes, one displaying a sine wave and
the other a cosine wave. The first call to subplot divides the figure
into two plotting areas (two rows and one column) and makes the first plot
area active. The grid of plot areas created by `subplot` is
numbered in column-major order (top to bottom, left to right).

— Function File: **subplot** (rows, cols, index)  
— Function File: **subplot** (rcn)  
> Set up a plot grid with cols by rows subwindows and plot
> in location given by index.
>
> If only one argument is supplied, then it must be a three digit value
> specifying the location in digits 1 (rows) and 2 (columns) and the plot
> index in digit 3.
>
> The plot index runs row-wise. First all the columns in a row are filled
> and then the next row is filled.
>
> For example, a plot with 2 by 3 grid will have plot indices running as
> follows:
>
> ```
> ```
>                +-----+-----+-----+
>                |  1  |  2  |  3  |
>                +-----+-----+-----+
>                |  4  |  5  |  6  |
>                +-----+-----+-----+
> ```
> ```
>
>
>
>
> **See also:** plot.
